# Optimizing risk stratification in pediatric febrile urinary tract infection: A single-center study in Japan

**DOI:** 10.1371/journal.pone.0335743

**Published:** 2025-11-03

**Authors:** Tomohiro Inoguchi, Riku Hamada, Yooka Nam, Chikako Terano, Ryoko Harada, Satoshi Narumi, Yuko Hamasaki, Kenji Ishikura, Masataka Honda, Hiroshi Hataya

**Affiliations:** 1 Department of Nephrology and Rheumatology, Tokyo Metropolitan Children’s Medical Center, Tokyo, Japan; 2 Department of Pediatrics, Keio University School of Medicine, Tokyo, Japan; 3 Department of Pediatrics, Kitasato University School of Medicine, Kanagawa, Japan; 4 Department of Nephrology, Faculty of Medicine, Toho University, Tokyo, Japan; West Bengal University of Animal and Fishery Sciences, INDIA

## Abstract

Selecting optimal therapeutic interventions for febrile urinary tract infection (f-UTI) is crucial to prevent complications such as kidney scarring. While current clinical guidelines provide risk-stratified imaging recommendations, they are largely based on Western populations and lack specific predictors for which children will ultimately require therapeutic interventions. This study aimed to establish risk stratification criteria for East Asian children with first-episode f-UTI. This retrospective single-center study analyzed patients aged 2–24 months with first-episode f-UTI. All patients underwent a standardized diagnostic and management protocol, including kidney–bladder ultrasound (KBUS) and voiding cystourethrography (VCUG), to ensure uniform evaluation. The primary outcome was “requirement for therapeutic intervention,” defined as one or more of the following: (1) urological surgery (2) antimicrobial prophylaxis (for vesicoureteral reflux grade ≥III) and (3) antimicrobial treatment for recurrent f-UTI. Multivariate logistic regression was performed to identify independent predictors associated with the interventions. A total of 216 patients were included (median age: 4 months). Overall, 59 patients required therapeutic interventions. Non-*Escherichia coli* infection (OR 3.3, 95% CI 1.3–8.7) and abnormal KBUS findings (OR 5.3, 95% CI 2.7–10.6) were identified as independent predictors. The sensitivity and specificity of the factors for predicting therapeutic intervention were 64.4% and 73.2%, respectively. This study identified non-*E. coli* infection and abnormal KBUS findings as key predictors for therapeutic interventions in East Asian children with first-episode f-UTI. These findings suggest that a more targeted approach based on these factors may optimize risk stratification and patient selection for VCUG, improving clinical decision-making.

## Introduction

Urinary tract infections (UTIs) are common among children [1]. During the first 8 years of life, approximately 7–8% of girls and 2% of boys develop UTIs [1]. In children, recurrent febrile UTIs (f-UTIs) lead to kidney scarring and contribute to kidney impairment [2]. Patients with urinary tract abnormalities, such as vesicoureteral reflux (VUR), may have recurrent f-UTIs. Clinicians usually perform extensive imaging studies, especially voiding cystourethrography (VCUG), to identify urinary tract abnormalities and determine the appropriate timing for therapeutic interventions, such as prophylactic antimicrobials and urologic surgery. However, VCUG has several disadvantages, including discomfort, radiation exposure, and high medical expenditure.

Clinical practice guidelines presented by the National Institute for Health and Care Excellence (NICE) and the American Academy for Pediatrics (AAP) are both widely referenced internationally. They provide recommendations for imaging studies after f-UTI using a risk stratification approach that balances the potential for kidney scarring with the invasiveness of diagnostic procedures [3,4]. While these guidelines provide criteria for imaging studies, they do not offer specific predictors of which children ultimately require therapeutic interventions. Furthermore, these guidelines are based primarily on Western populations and may not be directly applicable to East Asian children. This issue is particularly relevant in East Asian children, where differences in patient demographics, clinical practice patterns, and cultural factors—such as the low prevalence of circumcision—limit the direct applicability of international guidelines. Given these limitations, there is a critical need to establish evidence-based risk stratification criteria tailored to East Asian children. This study aimed to identify the clinical characteristics of East Asian children with first-episode f-UTI who required therapeutic interventions, including surgery and antimicrobial prevention/treatment for f-UTI recurrence.

## Materials and methods

We conducted a retrospective chart review of children aged 2–24 months presenting with their first episode of febrile urinary tract infection (f-UTI) at Tokyo Metropolitan Children’s Medical Center, Japan.

All procedures were performed in accordance with the ethical principles of the Declaration of Helsinki; the 2013 Ethical Guidelines for Epidemiological Studies issued by the Ministry of Health, Labour and Welfare of Japan; and the ethics board of the Tokyo Metropolitan Children’s Medical Center (approval number 2023b-39).

As all data were retrospective and based on information from patients’ medical records, the requirement for informed consent was waived according to the above guidelines.

All patients’ parents or guardians were given the opportunity to refuse to participate in the study. The study protocol was displayed publicly on the website of the Tokyo Metropolitan Children’s Medical Center in accordance with the above guidelines.

Data were accessed for research purposes from the hospital’s electronic medical records from 25/07/2023–30/10/2023, following approval by the institutional ethics board. Identifiable patient information was accessible to the investigators during the data collection phase; however, all data were anonymized before analysis, and no identifying information was used in the analysis or shared among the research team.

We included patients aged 2–24 months who were treated for first f-UTI and received both kidney–bladder ultrasound (KBUS) and VCUG between May 2010 and April 2018. F-UTI was defined as fever ≥38°C with ≥10^4^ colony-forming units (CFU)/mL or ≥10^5^ CFU/mL of an organism in urine collected through catheterization or clean-catch samples, respectively. Patients were excluded if they had less than 6 months of follow-up or were diagnosed with catheter-associated f-UTI. We defined patients as “requiring therapeutic interventions” if they met one or more of the following criteria: (1) received antimicrobial prophylaxis for VUR grade ≥III, (2) received antimicrobial treatment for recurrent f-UTIs or (3) received urological surgery. Antimicrobial prophylaxis for recurrent f-UTI was indicated only for patients with VUR Gr ≥ III in this study.

Patient demographics, clinical history, laboratory data, and imaging studies were collected retrospectively from medical records. Serum creatinine-based estimated glomerular filtration rate was calculated using a polynomial formula for Japanese children and adolescents [5].

**Fig 1:**
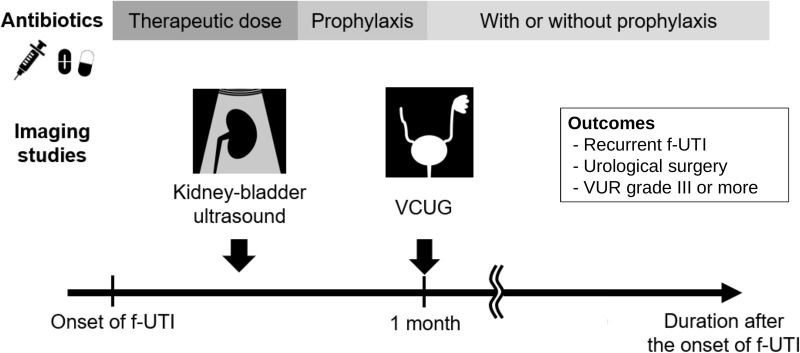
Standardized imaging and management protocol of first febrile urinary tract infection in our facility. All patients received therapeutic doses of antibiotics followed by prophylactic doses until voiding cystourethrography (VCUG) results were available. Kidney–bladder ultrasound (KBUS) was performed within a few days of the onset of f-UTI. VCUG was conducted approximately one month after the onset of f-UTI.

Imaging studies were performed according to the predefined standardized protocol (Fig 1). KBUS and VCUG were performed on all patients. We conducted KBUS within a few days after the onset of f-UTI. Abnormal findings on KBUS were defined as any anomalies of the kidney and urinary tract, not just hydronephrosis or ureteral dilation. VCUG was conducted approximately 1 month after the onset of f-UTI. VUR was graded according to the criteria determined by the International Reflux Study in Children [6]. In our study, a higher grade was used for the classification if the patient had bilateral VUR. KBUS was initially interpreted by a radiologist according to a standardized institutional protocol, and the results were subsequently evaluated by nephrologists. Abnormal findings were identified according to institutional criteria, incorporating established guidelines where applicable. VCUG was primarily interpreted by nephrologists, with consultation from urologists when necessary. VCUG interpretation was not blinded to KBUS findings, as the interpreting physicians had access to patients’ clinical information and prior imaging results, including KBUS. Both KBUS and VCUG findings were regularly reviewed in conferences to establish a unified interpretation.

Based on the protocol (Fig 1) patients received therapeutic doses of antibiotics followed by prophylactic doses until VCUG results were available. Maintenance antimicrobial prophylaxis was administered to patients with a VUR grade of ≥III. Urological surgery was performed for patients with urinary tract abnormalities for whom urologists judged that surgical treatment would be effective. Internal laxatives and glycerin enemas for constipation as well as topical steroids for the foreskin were administered at the discretion of the attending physician.

### Statistical analysis

Continuous variables were expressed as medians and interquartile ranges (IQR). To investigate the clinical features of patients requiring therapeutic interventions, we used univariate and multivariate logistic regression models, which were prepared to estimate the risk of therapeutic interventions associated with potential predictors such as bacteremia, causative organisms in the urine specimen, elevated creatinine levels, duration of fever after antibiotic initiation, and abnormal findings on KBUS. The inclusion of variables in the models was based on the clinical features indicated by the NICE and AAP guidelines [3,4]. All P-values were two-sided, with P < 0.05 considered significant. All statistical analyses were performed using EZR version 4.2.2 [7].

## Results

**Fig 2:**
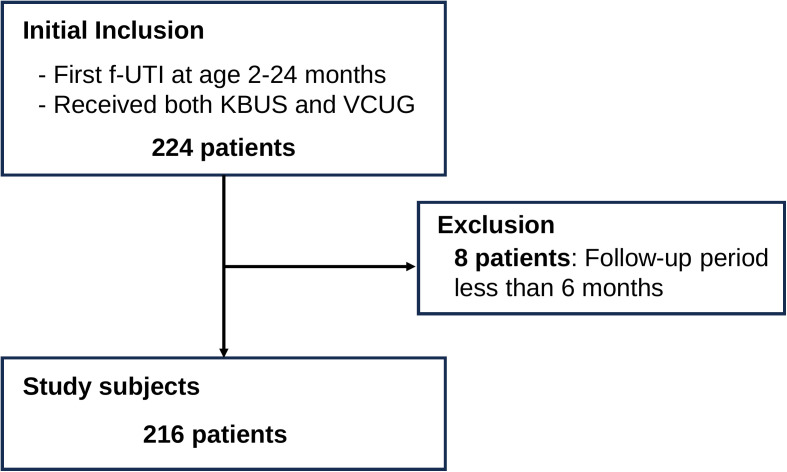
Study population flow diagram. Of 224 children with a first febrile urinary tract infection (f-UTI) who underwent both KBUS and VCUG, 8 were excluded (follow-up < 6 months), leaving 216 for the final analysis.

**Fig 3:**
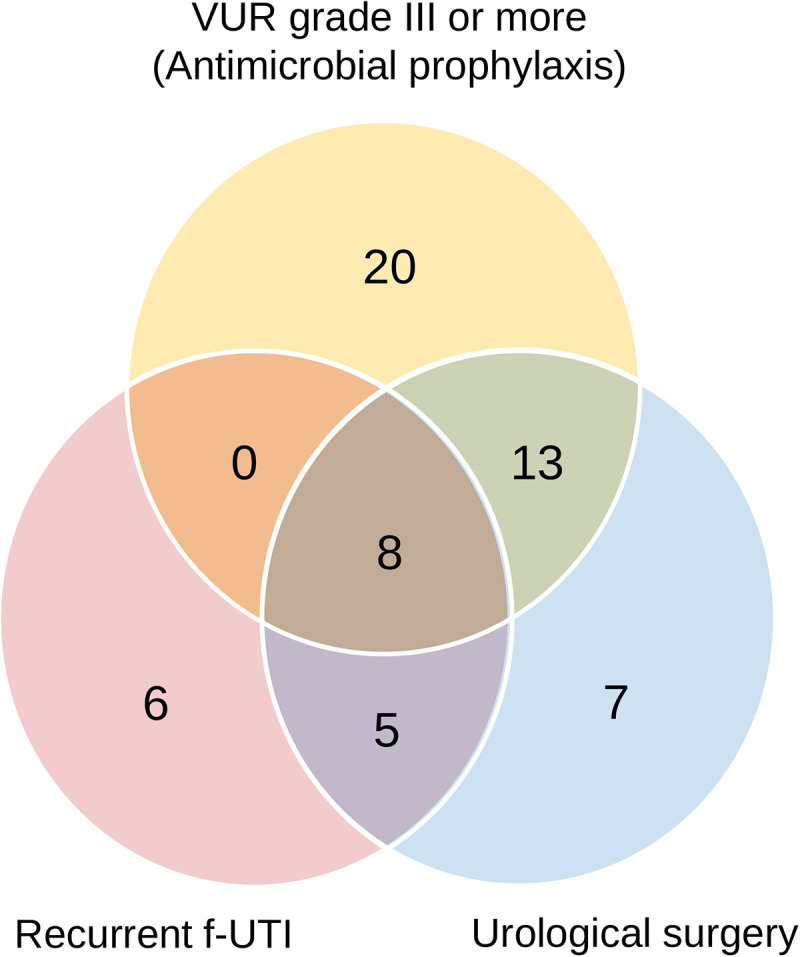
Overlap of therapeutic interventions. The Venn diagram shows patients who received antimicrobial prophylaxis for VUR grade ≥ III (n = 41), urologic surgery (n = 33), and/or antimicrobial treatment for recurrent f-UTI (n = 19). Intersections indicate patients who underwent multiple interventions.

Overall, 216 children (147 boys; 69 girls) were enrolled (Fig 2). All patients were Asian. All boys were uncircumcised, and 57.8% of boys were treated with topical steroids on the foreskin. Characteristics of the study subjects are summarized in Table 1. The median age at onset of f-UTI was 4 months (IQR, 2–7 months). Boys aged <12 months accounted for 65.3% of the participants. The median follow-up period was 37 months (IQR, 19–64 months). *Escherichia coli* (*E. coli*) was cultured in 88.4% of the urine specimens (S1 Table). Bacteremia was noted in six patients (2.8%). Abnormal KBUS findings were observed in 64 patients (29.6%), and the most common abnormality was ureteral dilation and hydronephrosis (S2 Table). VUR was observed in 56 patients (25.9%) including 41 (19.0%) with grade ≥III. Those 41 patients were subject to antimicrobial prophylaxis. Urethral strictures were observed in 19.4%. Recurrent f-UTI was observed in 19 patients. Urological surgery was performed in 33 patients (15.3%) (S3 Table), including transurethral resection or urethral calf ablation (N = 21) and ureteroneocystostomy (N = 13). Pyeloplasty for ureteropelvic junction stenosis was performed in three patients with hydronephrosis on KBUS. Collectively, our study identified 41, 33, and 19 patients who received antimicrobial prophylaxis, surgery, and treatment for recurrent f-UTIs, respectively. There was overlap among the three interventions, with 59 patients (27.3%) requiring interventions overall (Fig 3).

**Table 1:** Patient characteristics.

Characteristics	
Male, n (%)	147 (68.1)
Age at onset of f-UTI (months), median (IQR)	4 (2-7)
Age and sex group, n (%)	
Male aged <12 months	141 (65.4)
Male aged ≥12 months	6 (2.7)
Female aged <12 months	57 (26.4)
Female aged ≥12 months	12 (5.6)
Follow-up period (months), median (IQR)	37 (19-64)
Causative organisms on urine culture, n (%)	
*E. coli*	191 (88.4)
non-*E. coli*	25 (11.6)
Abnormal findings on KBUS, n (%)	64 (29.6)
VUR, n (%)	56 (25.9)
Grade I	9 (4.2)
Grade II	6 (2.8)
Grade III	19 (8.8)
Grade IV	19 (8.8)
Grade V	3 (1.4)
Bacteremia, n (%)	6 (2.8)
Kidney dysfunction (eGFR < 60 ml/min/1.73m^2^), n (%)	2 (0.9)
Persistent fever after indication of Abx, n (%)	7 (3.2)
Urological surgery, n (%)	33 (15.2)
Recurrent f-UTIs, n (%)	19 (8.8)

Abx: antibiotics; E. coli: Escherichia coli; eGFR: estimated glomerular filtration rate; f-UTI: febrile urinary tract infection; IQR: interquartile range; KBUS: kidney-bladder ultrasound; VUR: vesicoureteral reflux.

**Fig 4:**
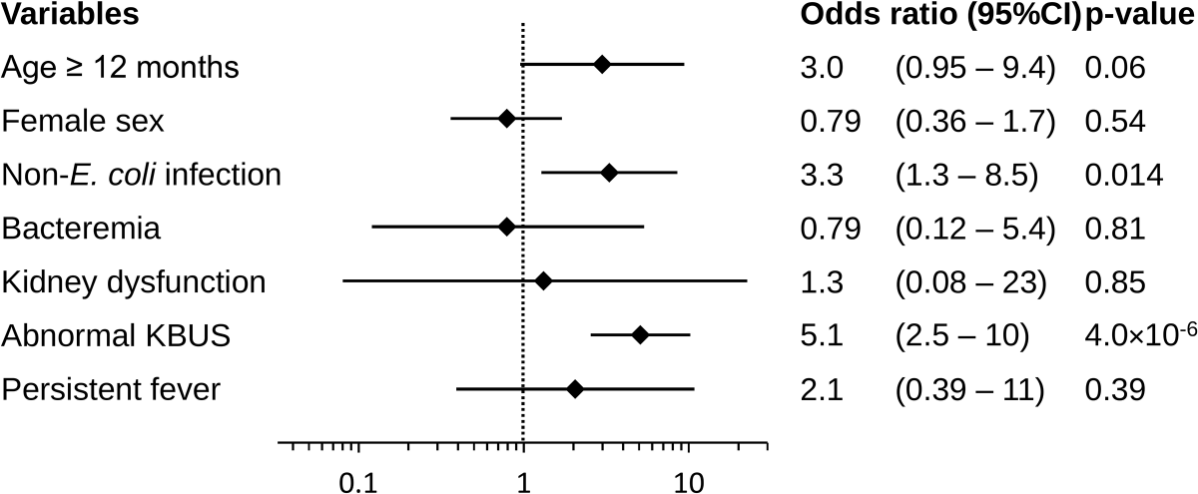
Multivariate logistic regression: predictors of later therapeutic intervention. Forest plot of odds ratios (OR) with 95% confidence intervals (CI) for factors entered into the logistic regression model. Non-*Escherichia coli* (*E. coli*) infection (OR 3.3, 95% CI 1.3–8.7, p = 0.014) and abnormal KBUS findings (OR 5.3, 95% CI 2.7–10.6, p < 0.001) were significantly associated with later intervention.

Univariate and multivariate logistic analyses were performed to determine the independent factors associated with therapeutic interventions. On univariate logistic regression analysis, non-*E. coli* infection and abnormal findings on KBUS were significant factors for therapeutic interventions (S4 Table). On multivariate logistic regression analysis, non-*E. coli* infection (OR: 3.3; 95% CI: 1.3–8.7, p = 0.014) and abnormal findings on KBUS (OR: 5.3; 95% CI: 2.7–10.6, p < 0.001) were significant factors for therapeutic interventions (Fig 4). When the composite outcome was broken down by component, non-*E. coli* infection remained significantly associated only with surgical intervention (OR: 3.0; 95% CI: 1.1–8.5, p = 0.035). In contrast, abnormal KBUS was consistently associated with all three outcomes––recurrence (OR: 5.6; 95% CI: 1.9–16.1, p = 0.0016), surgical intervention (OR: 4.0; 95% CI: 1.8–9.2, p < 0.001), and high-grade VUR (OR: 6.8; 95% CI: 3.1–15.0, p < 0.001) (S5 Table).

In our cohort, 80 patients (37%) had non-*E. coli* infection and abnormal findings on KBUS. If we restricted indication of VCUG for those 80 patients, the sensitivity, specificity, positive predictive value (PPV), and negative predictive value (NPV) for predicting patients requiring therapeutic intervention were 64%, 73%, 48%, and 85%, respectively.

Similarly, when risk groups were defined according to the NICE and AAP guidelines, 66 and 64 patients, respectively, were classified as candidates for VCUG. If VCUG had been limited to these 66 and 64 patients, the sensitivity, specificity, PPV, and NPV for predicting cases that required therapeutic intervention would have been calculated as 51%, 77%, 46%, and 81% based on the NICE guideline, and 54%, 80%, 50%, and 82% based on the AAP guideline (S1 Fig). The number of missed cases requiring therapeutic intervention due to the restriction of VCUG indications was estimated to be 21, 27, and 29 using our risk-based approach (non–*E. coli* infection or abnormal KBUS findings), the AAP guideline, and the NICE guideline, respectively (S2 Fig).

## Discussion

In this study, we aimed to identify the characteristics of f-UTI patients who later required therapeutic intervention(s), as a first step toward establishing evidence-based risk stratification criteria tailored to East Asian children. We identified two factors associated with therapeutic intervention: non-*E. coli* infection and abnormal KUBS findings. This study was conducted at a single center, where all first-episode f-UTI patients underwent a standardized diagnostic and management protocol, ensuring comprehensive and uniform evaluation. This approach minimized bias associated with variability in clinical assessment and allowed for a consistent comparison of the criteria (*i.e.,* NICE guideline, AAP guideline and ours) to determine which patients require therapeutic intervention.

A unique feature of our study is to establish clinical factors associated with later therapeutic interventions based on long-term follow-up. This is in contrast to most previous studies of f-UTIs, which used high-grade dilating VUR as a primary endpoint [8,9]. In this study, there were patients without high-grade VUR who received urologic surgery or antimicrobial treatment for recurrent f-UTIs during the median follow-up period of 37 months. Leveraging the long-term follow-up data, we defined therapeutic intervention as a composite endpoint to provide a more clinically relevant framework for the initial management and future risk stratification of first-episode f-UTI.

Our criteria for high-risk f-UTI––(i) non-*E. coli* infection or (ii) abnormal KBUS findings––resulted in a wider range of patients being defined as high-risk than in the NICE and AAP guidelines. This resulted in an improvement of sensitivity for predicting later requirement for therapeutic interventions by more than 10% (S1 Fig). The NICE and AAP guidelines recommend VCUG in specific cases: NICE guideline recommends VCUG for selected cases based on clinical and imaging findings, particularly in infants aged <6 months with atypical UTI or abnormal ultrasound findings, and in children aged 6 months to 3 years with poor urine flow, non–*E. coli* infection, family history of VUR, or dilatation on ultrasound associated with atypical UTI [4]. AAP guidelines recommend VCUG for atypical or complex cases, hydronephrosis, renal scarring, or findings suggestive of high-grade VUR or obstructive uropathy on ultrasound [3]. Both guidelines advocate risk stratification based on clinical presentation to restrict VCUG indications. However, this restriction raises concerns about missing high-risk cases requiring therapeutic intervention [10]. As shown in S2 Fig, our risk stratification approach missed fewer cases requiring therapeutic intervention compared to the AAP and NICE guideline-based VCUG indications. Under the NICE guideline-based VCUG indications, more cases with VUR grade ≥III were particularly missed. This is likely due to overly restrictive criteria for performing KBUS in infants younger than 6 months. In contrast, under the AAP guideline-based VCUG indications, patients required urological surgery were often missed, despite KBUS being recommended for all cases. This approach may fail to identify posterior urethral valves, where KBUS findings are often subtle.

The clinical presentation of f-UTI varies across regions owing to differences in patient demographics, ethnicity, and cultural practices. This study provides real-world data from East Asia, offering valuable insights into the epidemiology of pediatric UTI in this population. A notable characteristic of our study population was the predominance of male infants aged <12 months (65.3%), which is consistent with previous studies from Japan [11,12]. In Western countries, the prevalence of f-UTI in infants has not showed a significant sex difference [13,14]. One potential explanation is differences in circumcision practices. Neonatal circumcision is frequent in Western countries but is rare in Japan and other East Asian countries. Therefore, physiological phimosis, a risk factor for f-UTI in boys, is found at a higher frequency in boys born in East Asia [14,15]. A customized approach to the management of pediatric f-UTIs will need to take into account these regional differences in lifestyle.

Our study has some limitations. First, this was a single-center retrospective study, which limits the generalizability of our findings. Second, the sample size was relatively small, potentially limiting the statistical power to detect relatively weak predictive factors. Third, the criteria for surgical interventions, application of topical steroids for the foreskin, and treatment of constipation were left to the discretion of the physician, introducing potential variability in clinical decision-making. Fourth, because VCUG interpretation was not blinded to KBUS findings, interpretation bias may have been introduced; however, this reflects real-world clinical practice in our setting. Finally, there were no evaluations of long-term kidney function. Prospective, multi-center studies will be warranted to validate our findings and further refine the management of pediatric f-UTIs.

## Conclusions

This study identified the predicting factors (non-*E. coli* infections and abnormal KBUS findings) associated with therapeutic interventions in East Asian children with first-episode f-UTI. Selecting patients for VCUG based on our risk stratification criteria, rather than following the NICE and AAP guidelines, may be more effective in identifying patients requiring therapeutic interventions, particularly in Japanese children and populations with similar social and racial backgrounds.

## Supporting information

S1 Fig Comparison of VCUG indication algorithms: Risk stratification criteria and predictive performance for therapeutic intervention. Decision-tree diagrams show how this study’s criteria, the 2011 American Academy of Pediatrics (AAP) guideline, and the 2007 National Institute for Health and Care Excellence (NICE) guideline classify patients after initial clinical assessment and KBUS. The numbers indicate the number of patients in each branch; the “high-risk” arm identifies those selected for VCUG under each strategy. The right-hand table summarizes the diagnostic performance (sensitivity, specificity, PPV) of each approach for identifying children requiring therapeutic intervention.
(TIFF)

S2 Fig Patients requiring therapeutic intervention that would have been missed under different VCUG indication strategies. The Venn diagram illustrates the distribution of therapeutic outcomes (VUR grade ≥ III, recurrent f-UTI, and urological surgery) among patients who would have been missed if VCUG had been limited to each strategy’s high-risk group. Compared with this study’s criteria (21 missed cases), the AAP and NICE guidelines would have missed 27 and 29 cases.
(TIFF)

S1 Table Causative organisms on urine culture. (DOCX)

S2 Table Abnormal findings on kidney-bladder ultrasound in 64 patients. (DOCX)

S3 Table Forms of urological surgery. (DOCX)

S4 Table Univariate logistic regression of therapeutic intervention on variables. (DOCX)

S5 Table Multivariate logistic regression results for recurrence, surgical intervention, and high-grade VUR. (DOCX)
