# Supplementary figures and images for: Optimizing risk stratification in pediatric febrile urinary tract infection: A single-center study in Japan
Source: PLoS One. 2025 Nov 3;20(11):e0335743. doi: 10.1371/journal.pone.0335743 (PMC12582461; doi:10.1371/journal.pone.0335743)

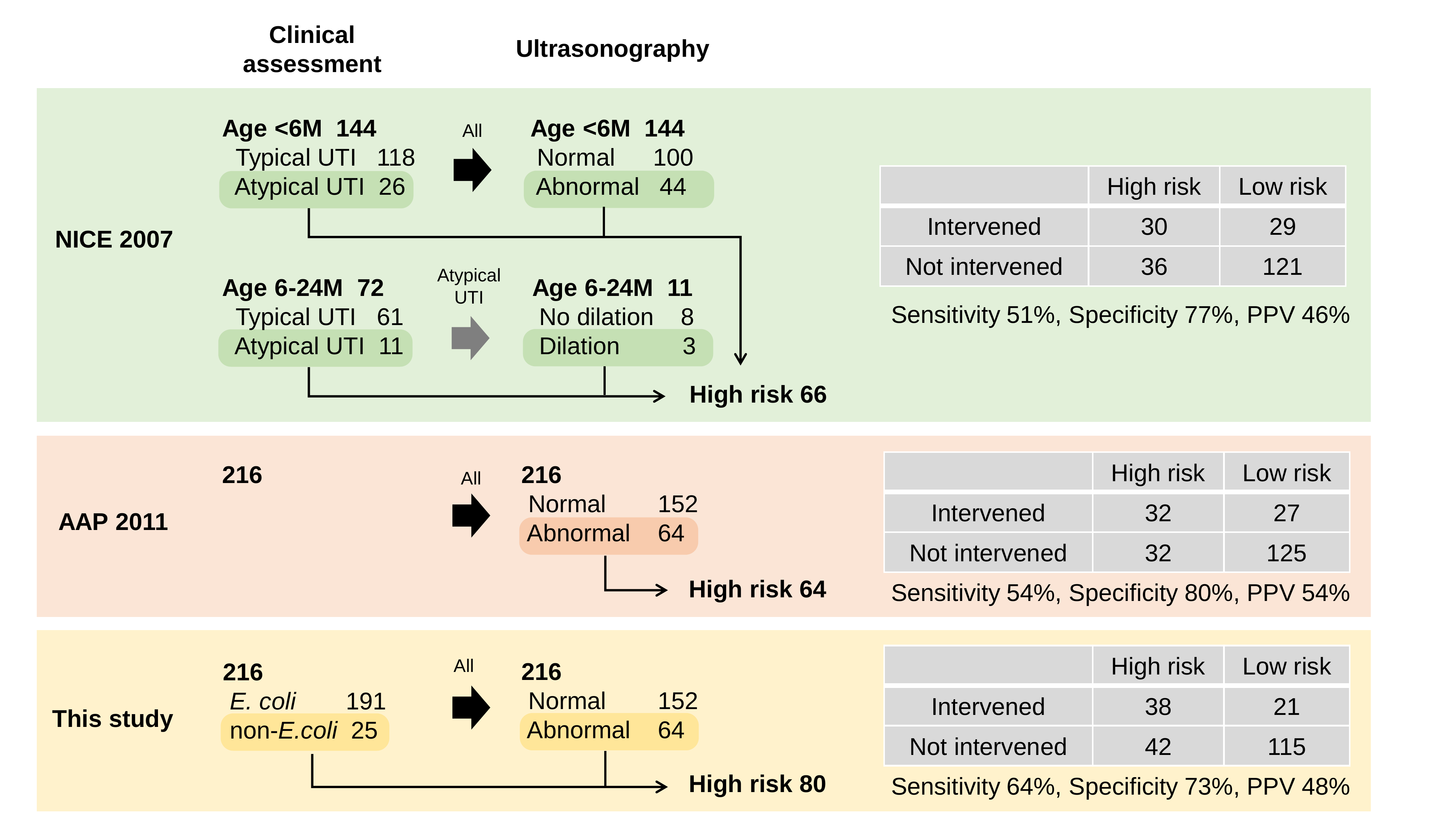

Supplement: S1 Fig — Decision-tree diagrams show how this study’s criteria, the 2011 American Academy of Pediatrics (AAP) guideline, and the 2007 National Institute for Health and Care Excellence (NICE) guideline classify patients after initial clinical assessment and KBUS. The numbers indicate the number of patients in each branch; the “high-risk” arm identifies those selected for VCUG under each strategy. The right-hand table summarizes the diagnostic performance (sensitivity, specificity, PPV) of each approach for identifying children requiring therapeutic intervention. (TIFF) [file pone.0335743.s001.tiff]

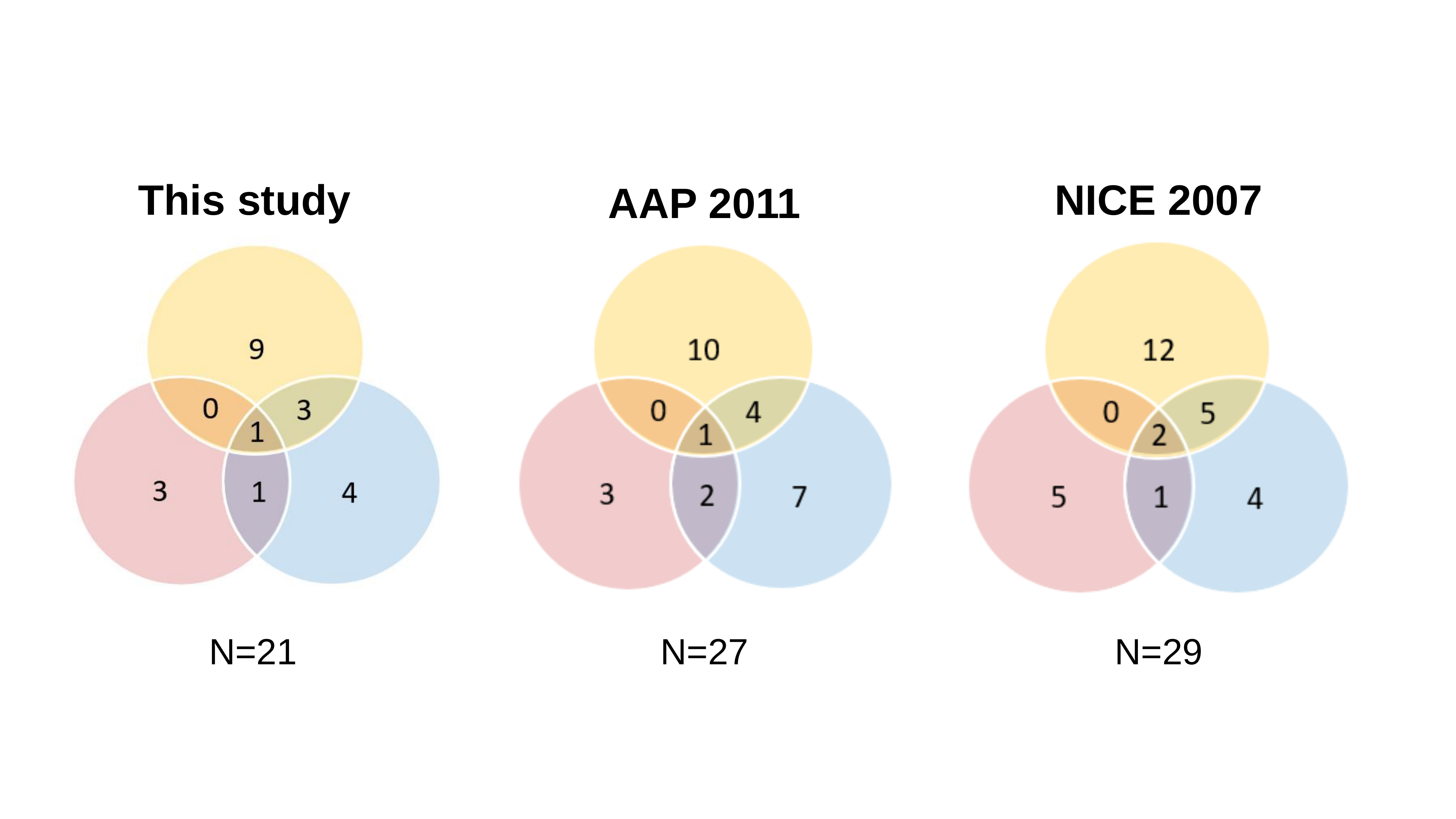

Supplement: S2 Fig — The Venn diagram illustrates the distribution of therapeutic outcomes (VUR grade ≥ III, recurrent f-UTI, and urological surgery) among patients who would have been missed if VCUG had been limited to each strategy’s high-risk group. Compared with this study’s criteria (21 missed cases), the AAP and NICE guidelines would have missed 27 and 29 cases. (TIFF) [file pone.0335743.s002.tiff]
