# Supplementary material for: Optimizing risk stratification in pediatric febrile urinary tract infection: A single-center study in Japan
Source: PLoS One. 2025 Nov 3;20(11):e0335743. doi: 10.1371/journal.pone.0335743 (PMC12582461; doi:10.1371/journal.pone.0335743)
Supplement: S1 Table — (DOCX) [file pone.0335743.s003.docx]

**S1 Table. Causative organisms on urine culture**

|  | n (%) |
| --- | --- |
| *Escherichia coli* | 191 (88.4) |
| *Enterococcus faecalis* | 9 (4.2) |
| *Klebsiella pneumoniae* | 6 (2.8) |
| *Klebsiella oxytoca* | 3 (1.4) |
| *Citrobacter freundii* | 3 (1.4) |
| Others | 4 (1.9) |
