# Supplementary material for: Optimizing risk stratification in pediatric febrile urinary tract infection: A single-center study in Japan
Source: PLoS One. 2025 Nov 3;20(11):e0335743. doi: 10.1371/journal.pone.0335743 (PMC12582461; doi:10.1371/journal.pone.0335743)
Supplement: S2 Table — (DOCX) [file pone.0335743.s004.docx]

**S2 Table.** **Abnormal findings on kidney-bladder ultrasound in 64 patients**

|  | n (%) |
| --- | --- |
| Dilation, hydronephrosis | 55 (25.4) |
| Horseshoe kidney | 3 (1.4) |
| Duplicated ureter | 3 (1.4) |
| Urolithiasis, nephrocalcinosis | 3 (1.4) |
| Hypoplastic kidney, solitary kidney | 3 (1.4) |
| Ureterocele | 3 (1.4) |
