# Supplementary material for: Optimizing risk stratification in pediatric febrile urinary tract infection: A single-center study in Japan
Source: PLoS One. 2025 Nov 3;20(11):e0335743. doi: 10.1371/journal.pone.0335743 (PMC12582461; doi:10.1371/journal.pone.0335743)
Supplement: S3 Table — (DOCX) [file pone.0335743.s005.docx]

**S3 Table. Forms of urological surgery**

|  |
| --- |
| Transurethral resection, urethral valve ablation |
| Ureteroneocystostomy |
| Pyeloplasty |
| Transurethral resection or incision |
| Urethroplasty |
| Deflux® injection treatment |
