# Supplementary material for: Optimizing risk stratification in pediatric febrile urinary tract infection: A single-center study in Japan
Source: PLoS One. 2025 Nov 3;20(11):e0335743. doi: 10.1371/journal.pone.0335743 (PMC12582461; doi:10.1371/journal.pone.0335743)
Supplement: S4 Table — (DOCX) [file pone.0335743.s006.docx]

**S4 Table.** **Univariate logistic regression of therapeutic intervention on variables**

| Variable | No. of patients | No. with  therapeutic intervention | OR  (95% CI) | p-value |
| --- | --- | --- | --- | --- |
| Age | | | | |
| ≥ 12 months | 18 | 7 | 1.78 (0.55–5.35) | 0.27 |
| <12 months | 198 | 52 | 1 (reference) |  |
| Sex | | | | |
| Female | 69 | 15 | 0.65 (0.31–1.32) | 0.25 |
| Male | 147 | 44 | 1 (reference) |  |
| Organism | | | | |
| Non-*E. coli* infection | 33 | 13 | 3.33 (1.33–8.77) | < 0.01* |
| *E. coli* infection | 183 | 46 | 1 (reference) |  |
| Bacteremia | | | | |
| Yes | 6 | 2 | 1.34 (0.12–9.64) | 0.67 |
| No | 208 | 57 | 1 (reference) |  |
| Kidney dysfunction (eGFR < 60 ml/min/1.73m^2^) | | | | |
| Yes | 2 | 1 | 2.68 (0.03–221.1) | 0.47 |
| No | 214 | 1 | 1 (reference) |  |
| Abnormal findings on KBUS | | | | |
| Yes | 64 | 32 | 4.59 (2.31–9.25) | < 0.01* |
| No | 152 | 27 | 1 (reference) |  |
| Persistent fever after indication of Abx | | | | |
| ≥ 48 hours | 7 | 3 | 2.1 (0.29–12.48) | 0.39 |
| < 48 hours | 209 | 56 | 1 (reference) |  |

ABx: antibiotics; CI: 95% confidence interval; E. coli; Escherichia coli; KBUS: kidney-bladder ultrasound; OR: odds ratio
