# Supplementary material for: Optimizing risk stratification in pediatric febrile urinary tract infection: A single-center study in Japan
Source: PLoS One. 2025 Nov 3;20(11):e0335743. doi: 10.1371/journal.pone.0335743 (PMC12582461; doi:10.1371/journal.pone.0335743)
Supplement: S5 Table — (DOCX) [file pone.0335743.s007.docx]

**S5 Table. Multivariate logistic regression results for recurrence, surgical intervention, and high-grade VUR**

|  | Recurrence | | Surgical  intervention | | High-grade  VUR | |
| --- | --- | --- | --- | --- | --- | --- |
| Variable | OR  (95% CI) | p-value | OR  (95% CI) | p-value | OR  (95% CI) | p-value |
| Age ≥ 12 months | 0.78  (0.088–6.81) | 0.82 | 2.65  (0.70–10.1) | 0.15 | 3.34  (0.93–12.0) | 0.15 |
| Female sex | 1.50  (0.45–4.74) | 0.49 | 0.82  (0.31–2.14) | 0.68 | 0.731  (0.30–1.79) | 0.68 |
| Non-*E. coli* infection | 1.75  (0.41–7.32) | 0.45 | 3.03  (1.08–8.53) | 0.035* | 1.46  (0.40–4.29) | 0.49 |
| Bacteremia | 3.93  (0.55–27.9) | 0.17 | 2.08  (0.31–14.2) | 0.45 | 0.98  (0.13–7.41) | 0.99 |
| Kidney dysfunction | 4.61  (0.26–82.7) | 0.30 | 3.58  (0.21–62.3) | 0.38 | 1.90  (0.11–32.7) | 0.65 |
| Abnormal KBUS | 5.55  (1.91–16.1) | < 0.01* | 4.03  (1.77–9.19) | < 0.01* | 6.81  (3.10–15.0) | < 0.01* |
| Persistent fever | 1.43  (0.12–17.3) | 0.78 | 2.07  (0.33–13.0) | 0.44 | 4.45  (0.79–.25.1) | 0.089 |
